# RADICAL VERSUS CONSERVATIVE METHODS IN ONE-STAGE PILONIDAL ABSCESS SURGERY: THE EXPERIENCE OF A TUNISIAN CENTER

**DOI:** 10.1590/0102-672020220002e1713

**Published:** 2022-12-19

**Authors:** Mohamed Fares Mahjoubi, Mehdi Ben-Latifa, Yasser Karoui, Bochra Rezgui, Amel Ben-Belaid, Nada Essid, Ali Ben-Ali, Mounir Ben-Moussa

**Affiliations:** 1 Charles Nicolle Hospital, Department of Surgery A – Tunis, Tunisia;; 2 University Tunis El Manar, Faculty of Medicine of Tunis – Tunis, Tunisia;; 3 Sahloul Hospital, Department of Surgery – Sousse, Tunisia;; 4 University of Sousse, Faculty of Medicine of Sousse – Sousse, Tunisia;; 5 Regional Hospital of Jendouba, Department of Surgery – Jendouba, Tunisia.

**Keywords:** Abscess, Pilonidal Sinus, Recurrence, Wound Healing, Abscesso, Seio Pilonidal, Recidiva, Cicatrização

## Abstract

**BACKGROUND:**

Surgical treatment for pilonidal abscess is the gold standard, but not yet well codified. Different techniques proposed can be conservative or radical.

**AIMS:**

The aim of our study was to compare postoperative outcomes of both methods in one-stage treatment strategy.

**METHODS:**

This is a comparative study including patients operated on for pilonidal abscess, with a satisfactory postoperative follow-up, over a period of 4 years. We looked for the occurrence of postoperative recurrence in the medical records or by interviewing reachable patients.

**RESULTS:**

We analyzed 57 patients: 33 males and 24 females. The mean age was 26.9±10 years. The type of operation was excision in 46 (81%) cases and incision in 11 (19%) cases associated with curettage in three cases and drainage in 1 case. There was no statistically significant relationship between the type of surgery and the occurrence of postoperative surgical complications (p=1) and hospital stay (p=0.4). Excision of pilonidal abscess was significantly associated with a longer time to return to activity (p=0.04). Conservative surgery was significantly associated with faster healing of the surgical wound (p<0.001). The recurrence rate was 19% in radical surgery and 54% in conservative surgery. Radical surgery was significantly associated with a lower recurrence rate than incision procedure (p=0.02).

**CONCLUSIONS:**

Excision of pilonidal abscess was the common technique in our series, with a significantly lower rate of recurrence of the disease than after incision. However, the long convalescence following excision and the longer operating time, particularly in an emergency context, may sometimes lead to choosing conservative surgery.

## RESUMO

**RACIONAL:**

O tratamento cirúrgico do abscesso pilonidal é a indicação habitual, mas ainda não está bem codificado. Diferentes técnicas propostas podem ser conservadoras ou radicais.

**OBJETIVOS:**

Comparar os resultados pós-operatórios de ambos os métodos, em uma única etapa da estratégia de tratamento.

**MÉTODOS:**

Estudo comparativo incluindo todos os pacientes operados por abscesso pilonidal, com um seguimento pós-operatório satisfatório, durante um período de 4 anos. Foi avaliada a ocorrência de recidiva pós-operatória nos prontuários médicos ou entrevistando pacientes.

**RESULTADOS:**

Foram incluídos no estudo 57 pacientes: 33 homens e 24 mulheres. A média de idade foi de 26,9±10 anos. O tipo de operação foi a excisão em 46 casos (81%) e a incisão em 11 casos (19%) associada à curetagem em três casos e a drenagem em um caso. A excisão do abscesso pilonidal foi significativamente associada a um tempo maior para retornar à atividade (p=0,04). A cirurgia conservadora foi significativamente associada a uma cicatrização mais rápida da ferida cirúrgica (p<0,001). A taxa de recidiva foi de 19% em cirurgia radical e de 54% em cirurgia conservadora. A cirurgia radical foi significativamente associada a uma taxa de recidiva menor do que o procedimento de incisão (p=0,02).

**CONCLUSÕES:**

A excisão do abscesso pilonidal foi a técnica comum nesta casuística, com taxa de recidiva da doença significativamente menor do que após a incisão. No entanto, a longa convalescença após a excisão e o maior tempo de operação, pode às vezes levar à escolha de uma cirurgia conservadora.

## INTRODUCTION

Pilonidal abscess is a common condition in young people. Its prevalence can reach up to 0.7% of the population^18^. It is the acute suppurative form of pilonidal disease^13^. It is true that pilonidal disease has been extensively treated in the literature, but the management of its abscessed form has not received sufficient attention. Moreover, surgical treatment, although being the gold standard, is not yet well codified^1^. A two-stage surgical treatment is often proposed. Different surgical techniques proposed can be grouped into conservative and radical techniques.

The aim of our study was to compare the two surgical methods used in one-stage treatment strategy, in terms of postoperative complications, hospital stay, time to return to normal activities, wound healing time, and recurrence of pilonidal disease.

## METHODS

This was a comparative study conducted in the Department of Surgery A of “Charles Nicolle” Hospital in Tunis over a period of 4 years, from January 2015 to December 2018, including all patients operated on for pilonidal abscess with a satisfactory postoperative follow-up. Patients treated with a two-stage treatment strategy were not included. Patients with missing data on medical records, patients lost to follow-up, and patients unable to reach through phone were excluded from the study.

We have defined any procedure of wide or limited excision of the abscess and infected tissues as radical surgery. We have defined any incision with pus evacuation, with or without curettage or drainage, as conservative surgery.

Clinical, paraclinical, and operative data were extracted from medical records and from operative reports. Some missing data were recovered by reaching patients by phone. Data entry and analysis were performed using the Statistical Package for Social Sciences (SPSS) software version 23.0. The p-value of <0.05 was considered statistically significant.

Our locally appointed Ethics Committee has approved the research protocol and informed consent has been obtained from the subjects.

## RESULTS

### Descriptive study

#### Preoperative data

A total of 57 patients were included in the study. There were 33 (58%) men and 24 (42%) women. The mean age was 26.9±10 years, with extremes of 15 and 60 years. Two patients were diabetic. About 61% of patients had a body mass index of >25 kg/m^2^. Twelve patients were operated before for pilonidal disease. The average duration of clinical signs was 5.6±3.2 days. The average abscess size was about 3.6±1.1 cm. According to biological findings, hyperleukocytosis was noted in 60% of patients and the C-reactive protein was increased in 61% of cases.

#### Operative findings

All patients were operated on: 55 patients under general anesthesia, 70% in lateral position and 30% in supine position. A total of 53 patients were operated on by a resident doctor. The type of operation was an excision procedure in 46 (81%) cases and an incision procedure in 11 (19%) cases associated with curettage in 3 cases and drainage in 1 case. The average operation time was 14.3±5.9 min, with extremes of 5 and 30 min.

#### Postoperative outcomes

A postoperative antibiotic therapy was prescribed in seven cases, based on amoxicillin-clavulanic acid, for a mean duration of 5.9±2.3 days. The average hospital stay was 1.1±0.4 days, with extremes of 1 and 4 days.

Only one patient presented a postoperative hemorrhage occurring after the excision procedure. There was no reported case of wound infection. The average time to return to normal activities was 21±13 days, with extremes of 2 and 90 days. The average healing time was 63.4±32.2 days, with extremes of 10 and 150 days.

Recurrence of pilonidal disease was noted in 26% (15 cases) of patients. The mean time of recurrence was 9.7±6.1 months, with extremes of 2 and 24 months.

### Analytic study

The analytic study findings are summarized in Table 1.

**Table 1:** Postoperative outcomes of radical and conservative procedures for pilonidal abscess.

Postoperative outcomes	Radical surgery (n=46)	Conservative surgery (n=11)	p-value
Postoperative complications	1	0	1
Hospital stay	1.1±0.4	1	0.2
Average time to resume normal activities	23.4±13.4	10.5±5.1	0.04
Average wound healing time	70.9±29.9	28.4±4.9	<0.001
Recurrence	9 (19%)	6 (54%)	0.02

Bold indicates statistically significant values.

There was no relationship between the type of surgery and the occurrence of postoperative surgical complications (p=1). The mean postoperative length of stay was 1 day for patients who had incision and 1.11 days for those who had excision. The type of procedure and the length of hospital stay were not statistically associated (p=0.4).

The mean time to return to activity was 10.5±5.1 days for patients who had an incision procedure and 23.4±13.4 days for patients who had an excision procedure. Excision of pilonidal abscess was significantly associated with a longer time to return to activity compared to incision (p=0.04). The mean time to wound healing was 28.4±14.9 days for patients who had conservative surgery and 70.9±29.9 days for patients who had radical surgery. Conservative surgery was significantly associated with faster healing of the surgical wound (p<0.001).

The recurrence rate was 19% in radical surgery group and 54% in conservative surgery group. Complete excision of pilonidal abscess was significantly associated with a lower recurrence rate than the incision procedure (p=0.02).

## DISCUSSION

Our study involved the following main findings: the comparison of conservative and radical surgical methods did not find any differences in terms of complications and postoperative hospital stay. Wound healing time and time to return to normal activities were shorter after conservative surgery. In contrast, radical surgery resulted in less recurrence of pilonidal disease.

Intervention type in pilonidal abscess is still a matter of controversy. Some authors currently recommend the incision of the abscessed pilonidal sinus, whether it is the first episode of abscess or a recurrence^12,19^. Hanley recommends an urgent incision of the abscess, followed by excision of the pilonidal sinus after 4–6 weeks^8^. However, excision methods, whether large or small, are also validated techniques for the treatment of pilonidal abscess^6^.

According to the habits of our center, a two-stage treatment was preferred neither by practitioners nor by patients. A one-stage treatment has the advantage of only one anesthesia and one procedure. This explains the more frequent recourse to radical surgical treatment, trying to solve the problem once and for all, at the cost of a longer procedure and a greater loss of substance.

The postoperative hospital stay depends not only on the surgical procedure but also on the type of anesthesia^17^. Naja et al. compared two groups of patients operated on for pilonidal disease: a group of patients operated on under general anesthesia and a group of patients operated on under local or locoregional anesthesia. They concluded that the group of patients operated on under local or locoregional anesthesia had a shorter postoperative hospital stay than the group of patients operated on under general anesthesia (p<0.001). No patient operated under local or regional anesthesia stayed more than 1 day in the hospital after surgery, while 10% of patients operated under general anesthesia required to stay more than 2 days in the hospital postoperatively. The use of opioid-containing opioids was higher in the group of patients operated on under general anesthesia^17^.

Our study did not evaluate the impact of anesthesia type, given the small number of patients in the local anesthesia group (only two patients were operated on under local anesthesia).

Postoperative surgical complications are generally of a hemorrhagic or suppurative nature^5^. Postoperative hemorrhage can complicate any surgery of the pilonidal sinus, particularly excisions. It occurs mainly in the immediate postoperative period (the first 48 h) and may require emergency hemostasis (in the patient's bed or in the operating theatre)^13^. Infection of the surgical wound can complicate any surgery of the pilonidal sinus whatever the procedure used. Its rate varies between 0 and 13%^13^.

The low rate of complications in our series could be explained by the fact that some complications could be diagnosed and treated without being noted in medical records.

Wound healing time after incision of a pilonidal abscess varies between different series from 10 to 112 days^20^. Table 2 summarizes the average wound healing time after incision in the different series^2,4,10,15,16,20^.

**Table 2:** Average wound healing time after incision in the different series.

Series	Average wound healing time
Webb et al.	63 days
Matter et al.	30 days
Jensen et al.	5 weeks
Bissett et al.	8.6 weeks
Courtney et al.	12–14 days
McLaren et al.	6 weeks
Our study	31 days

The wound healing time after excision of pilonidal abscess varies in different series from 1 to 3 months. Table 3 summarizes the average wound healing time after excision in the different series^8,9,15^.

**Table 3:** Average wound healing time after excision in the different series.

Series	Average wound healing time
Hosseini et al.	8 weeks
Matter et al.	30 days
Hanley et al.	6–12 weeks
Our study	71 days

In our series, the results we found are within the range of the results found by other authors.

The recurrence rate of pilonidal disease after surgery varies according to the used technique: it is about 40–50% in simple incision procedures that could be reduced to 15% if the incision is combined with curettage of the abscess cavity and drainage^1,13^.

The recurrence rate varies between different series from 1 to 36% after excision^1^. Table 4 summarizes the recurrence rate of pilonidal disease after excision in different series^3,7,11,14,21^. Our results are in line with the different results found in the literature.

**Table 4:** Recurrence rate of pilonidal disease after excision in different series.

Series	Recurrence rate (%)
Wood et al.	1
Golz et al. Kronborg et al.	6 13
Lamke et al.	15
Clothier et al.	36
Our study	19

## CONCLUSION

Excision of the pilonidal abscess was the most commonly used technique in our series, with a significantly lower rate of recurrence of the disease than after incision. However, the long convalescence following this method compared to the conservative method and the longer operating time, particularly in an emergency context, may sometimes lead to choosing conservative surgery. Therefore, we recommend complete excision as a reference treatment for pilonidal abscesses, except in the case of large abscesses for which an incision is preferable to get through the acute phase and secondarily adapt the therapeutic strategy.
